# Immunohistochemical markers and the clinical course of adenosarcoma: a series of seven cases

**DOI:** 10.1186/s13000-020-01036-5

**Published:** 2020-09-24

**Authors:** Makiko Omi, Akiko Tonooka, Tomohiro Chiba, Yuji Tanaka, Atsushi Fusegi, Yoichi Aoki, Hidetaka Nomura, Hiroyuki Kanao, Yutaka Takazawa

**Affiliations:** 1 Department of Gynecologic Oncology, Cancer Institute Hospital, Japanese Foundation for Cancer Research, 3-8-31 Ariake, Koutouku, Tokyo, 135-8550 Japan; 2 Division of Pathology, Cancer Institute Hospital, Japanese Foundation for Cancer Research, 2-2-2 Toranomon, Minatoku, Tokyo, 105-8470 Japan; 3 Department of Pathology, Toranomon Hospital, 2-2-2 Toranomon, Minatoku, Tokyo, 105-8470 Japan

**Keywords:** Mullerian adenosarcoma, Immunohistochemical staining, Sarcomatous overgrowth, ER/PR receptor, p53, SMARCA4, BCOR

## Abstract

**Background:**

Uterine adenosarcoma, a rare uterine tumor subtype, is a biphasic tumor consisting of epithelial and mesenchymal elements. To date, there is no research comparing the histopathological features and immunohistochemistry of primary and recurrent tumors; furthermore, the relationship between pathology and the clinical course remains unclear. We reviewed the pathology and immunohistochemical features of patients with adenosarcoma and investigated the relevance of the histomorphological features to the clinical course. We also compared the immunohistochemical features of the primary and recurrent tumors.

**Methods:**

The data of seven patients with adenosarcoma who underwent surgery in our hospital were evaluated. We performed immunohistochemical staining for the progesterone receptor, estrogen receptor, p53, and two Switch/Sucrose Non-Fermentable chromatin remodeling proteins (SMARCA4, BCOR), which were recently developed for the undifferentiated sarcoma diagnosis in addition to conventional staining methods.

**Results:**

All patients had International Federation of Gynecology and Obstetrics stage IB–IC diseases. All tumors were polypoid and every patient presented with abnormal uterine bleeding. Six patients aged over 50 years and were menopausal; one patient aged under 50 years and was non-menopausal (average age: 59.1 years). Histologically, the sarcomatous components were homologous and heterogenous in six and one patient, respectively. Four and three cases were recurrent and non-recurrent, respectively. The recurrent patients showed high-grade morphology with sarcomatous overgrowth and were negative for ER and PR. Three recurrences could be evaluated by imaging, showing recurrence only in a distant area; biopsy specimens from these tissues revealed the identical mesenchymal component found in the primary tumor without a benign epithelial component. Immunohistochemical staining results were also similar to the corresponding of the original tumor, except for the p53 expression in one patient. At the primary site, p53 was overexpressed in two recurrent patients and had a wild-type level in one recurrent patient; however, all three recurrent tissues showed p53 overexpression. None of our patients showed SMARCA4 loss, and BCOR expression was positive in one case.

**Conclusions:**

Initial pathological adenosarcoma analysis with appropriate immunohistochemical staining is vital for prognostic assessment. p53 expression might increase at recurrence. SMARCA4 and BCOR might not be an index of malignancy.

## Background

Adenosarcomas are tumors of low malignant potential, consisting of benign epithelial and malignant mesenchymal components. They are a rare subtype of uterine tumor, which represents less than 0.5% of uterine malignancies [1]. The median age at diagnosis is in the 50s, typically in postmenopausal women, but also occurs in adolescents to seniors, with age ranging from 13 to 94 years [2].

As they are rare, the pathology and immunohistochemical staining methods used for diagnosis and prognostic prediction are limited. Pathologically, some cases accompanied with sarcomatous overgrowth, deep myometrial invasion, extrauterine invasion, and/or heterogenous element, have poor prognoses [3–6]. Similar to an endometrial stromal sarcoma, immunohistochemical staining of the sarcoma components are highly positive for CD10, WT1, estrogen receptor (ER), and progesterone receptor (PR). In cases of sarcomatous overgrowth, the expression of these markers generally decreases, reflecting the dedifferentiation of the mesenchymal component [7, 8]. *TP53* pathway alterations are frequently found in high-grade adenosarcomas, resulting in nuclear atypia and severe pleomorphism identifiable at low-power magnification. Moreover, the p53 protein expression, detected by immunohistochemistry, is highly correlated with the mutation status. High-grade adenosarcomas with p53 protein overexpression harboring a *TP53* missense mutation or complete absence of expression harboring a *TP53* nonsense mutation are aggressive tumors with short-interval recurrences and metastases, regardless of sarcomatous overgrowth [9]. Genetic alterations in the Switch/Sucrose Non-Fermentable (SWI/SNF) chromatin remodeling complex components, such as SMARCA4 [10] and BCOR [11, 12], are recently reported in young high-grade uterine sarcomas, but their relationship with adenosarcomas remains unknown. We reviewed the pathology and immunohistochemical features of seven adenosarcoma cases and investigated the relevance of the histomorphological features to the clinical course. In addition, we compared the immunohistochemical features of primary and recurrent tumors; to the best of our knowledge, our study is the first to make this comparison.

## Material and methods

### Participant selection

We identified patients diagnosed pathologically with uterine adenosarcoma who were treated in our institute between the years 1992–2017. The specimen data and available clinical information were included.

The following clinical features were recorded: age at diagnosis, menopausal or not, symptoms, type of surgery, adjuvant chemotherapy, recurrence, site of recurrence, treatment after recurrence, follow-up period, and status at the last known visit. The following pathologic variables were recorded: International Federation of Gynecology and Obstetrics (FIGO) stage, presence of lymphovascular space invasion, mitotic activity, sarcomatous component (homologous/heterogenous), presence of sarcomatous overgrowth, and depth of myometrial invasion. Sarcomatous overgrowth was defined when the sarcomatous component occupies were more than 25% of the tumor volume. High-grade morphology was defined as a sarcoma with severe nuclear pleomorphism, prominent nucleoli, increased number of mitosis, and necrosis. The institutional review board (IRB) of the Cancer Institute hospital of JFCR approved this study (IRB approval No. 2020–1119). Informed consent was not required for this retrospective study.

### Immunohistochemistry

Primary antibodies and their dilutions are shown in Table 1. Cytoplasmic staining was considered positive for cytokeratin, desmin, SMA, CD10, CD34, and HHF34. Nuclear staining more than 50% was considered positive for ER, PR, MIB-1, cyclin D1, S100, SMARCA4, and BCOR. For p53, complete loss, focal nuclear expression, and nuclear expression in more than 90% of the tumor cell population were considered null (mutant type), normal (wild-type), and overexpression (mutant type), respectively.

**Table 1:** Immunohistochemical reagents and methods

Antibody	Clone	Dilution	Supplier
Cytokeratin	AE1 and AE3	1:200	Leica
CD34	NU-4A1	1:5	Nichirei
Desmin	DE-R-11	1:1000	Leica
SMA	1A4	1:1000	DAKO
CD10	56C6	1:100	Leica
Ki-67	Mib-1	1:200	DAKO
ER	SP1	Read to use	VENTANA (Roche)
PR	IE2	Read to use	VENTANA (Roche)
SMARCA4	ERP3921	1:50	Abcam, Cambridge, MA, USA
S100	Polyclonal	1:1000	Leica
Cyclin D1	P2D11F11	1:20	Leica
BCOR	C-10	1:100	SANTA CROZ
HHF35	HHF35	1:500	Enzo
P53	DO-7	1:500	DAKO

*SMA* smooth muscle antibody, *ER* estrogen receptor, *PR* progesterone receptor

## Results

### General clinical features of the patients (Table 2)

The average age at diagnosis was 59.1 years (range, 43–75 years). Six patients were menopausal. The follow-up period ranged from 2 to 114 months. All seven cases showed polypoid growth inside the uterine cavity, accompanied by abnormal bleeding. All patients underwent hysterectomy and bilateral oophorectomy, and three patients received an additional lymphadenectomy. Four patients underwent chemotherapy as adjuvant therapy. Four patients experienced recurrence, at 6, 10, 12, and 71 months after primary surgery. Three and one patients had distant recurrences, and an unknown site of recurrence, respectively. Two patients died soon after recurrence (within 1 and 2 months); one patient with pulmonary metastasis was cured, but died of another disease; one patient transferred to another hospital at recurrence.

**Table 2:** Clinical features of Mullerian adenosarcoma

Case	Age (years)	Menopause	Macroscopic findings	Surgery	Adjuvant therapy	PFS (month)	Site of recurrence	OS (month)	Status at last follow
Recurrent case									
1	50	Menopause	polypoid	mRH + BSO	IEP	6	Retroperitoneal tissue	7	DOD
2	57	Menopause	polypoid	mRH + BSO + PLA + PALA	–	71	Lung, Bone	73	DOD
3	66	Menopause	polypoid	TAH + BSO + PLA + PALA	–	12	Lung	31	Death of other disease (SAH)
4	65	Menopause	polypoid	TAH + BSO	–	10	Unknown site	10	AWD
Non-recurrent case									
5	75	Menopause	polypoid	TAH + BSO	5FU	2		2	NED
6	58	Menopause	polypoid	mRH + BSO + PLA + PALA	5FU	110		110	NED
7	43	–	polypoid	TAH + BSO + PLA	IAP	114		114	NED

*BSO* bilateral salpingo-oophrectomy, *DOD* death of disease, *IAP*: Ifosfamide and doxorubicin and cisplatin, *IEP* ifosfamide and epirubicin and cisplatin,

*5-FU* 5-furuolouracil, *mRH* modified radical hysterectomy, *NED* no evidence of disease, *PALA* paraaortic lymphadnectomy, *PLA* pelvic lymphadnectomy,

*SAH* subarachnoid hemorrhage

### Pathological features and immunohistochemical stains (Tables 3 and 4)

**Fig. 1:**
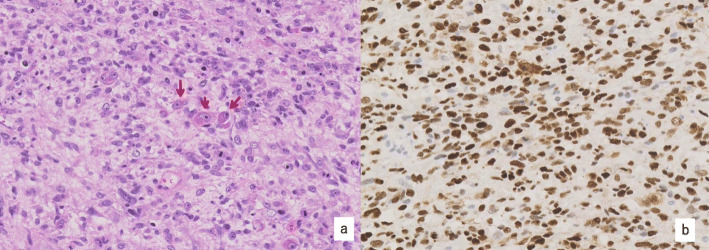
Case 4. H&E staining (original magnification × 400). Rhabdoid tumor cells with eosinophilic cytoplasm. H&E: hematoxylin and eosin

All patients had FIGO stage I disease with myometrial invasion; four and three patients had stage IB and IC diseases, respectively. Sarcomatous components were homologous and heterogenous (rhabdomyoblasts. Fig. 1) in six and one patients, respectively. Two patients had lymphovascular space invasion. Five patients (four patients with recurrence and one patient with a primary tumor) had high-grade adenosarcoma with mitosis more than 5/10 high power field (HPF), with sarcomatous overgrowth, and were negative for ER and PR. Three recurrent patients had an MIB-1 proliferation index over 50%. In the primary tumor, two and one recurrent patients showed overexpression and wild-type expression of p53, respectively; however, they all showed overexpression of p53 at the recurrent tissue. None of the patients showed SMARCA4 loss; one patient was positive for BCOR (80% of tumor cells).

**Table 3:** Pathological features and Immunohistochemical stains (Primary site)

Case	FIGO stage	LVSI (Ly V)	Grade	Mytosis /10 HPF	Sarcomatous component	SO	Myoinvasion	SMARCA4	BCOR	ER	PR	CD10	P53	MIB-1 (%)	Cyclin D1	Cytokeratin	Desmin	SMA	other
Recurrent cases																			
1	IC	+ +	High	5	homologous	+	> 1	+	–	–	–	–	Over express	70	–	–	–	–	CD34(−) S100(−)
2	IB	–	High	10–15	homologous	+	< 1/2	+	+	–	–	+	Wild type	50	+	–	–	–	HHF35(−) CD34(−) S100(−)
3	IB	–	High	10	homologous	+	< 1/2	+	–	–	–	+	Over express	50	–	ND	+	+	HHF35(+)
4	IC	+ −	High	30	heterogenous	+	> 1/2	+	ND	–	–	ND	Over express	ND	–	ND	ND	ND	
Non-recurrent cases																			
5	IB	–	High	5	homologous	+	< 1/2	+	–	–	–	+	Wild type	30	+	–	+	ND	
6	IB	–	Low	3	homologous	–	< 1/2	+	–	+	+	–	Wild type	5	–	ND	–	ND	
7	IC	–	Low	2	homologous	–	> 1/2	+	ND	+	+	ND	Null	ND	+	ND	ND	ND	

*LVSI* lymphovascular invasion, *ND* not done, *SO* salcomaous overgrowth

**Table 4:** Pathological features and Immunohistochemical stains (Recurrent site)

Case	ER	PR	CD10	P53	MIB-1(%)	SMA	other
1	–	–	–	Over express	80	–	
2	–	–	+	Over express	50	–	Desmin(−) HHF35(−) Cytokeratin(−) S100(−) CD34(−)
3	–	–	+	Over express	50	ND	Desmin(+)

**Fig. 2:**
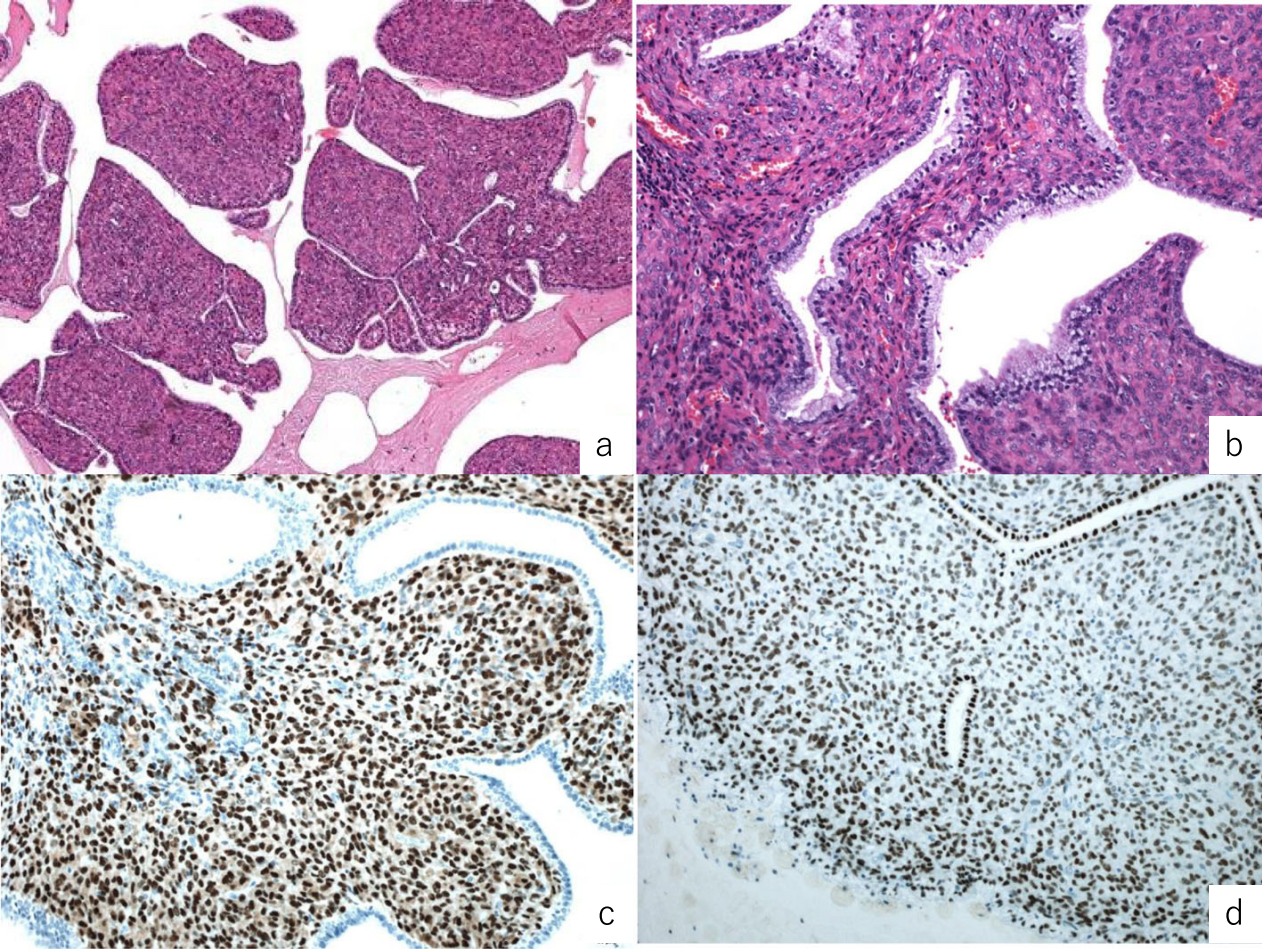
Case 6. (**a**) H&E staining (original magnification × 100), (**b**) H&E staining (original magnification × 400). A phyllodes-like structure composed of mild atypical cells covered by non-atypical epithelial metaplastic cells can be observed. H&E staining is positive for (**c**) ER and (**d**) PR (original magnification × 400). ER: estrogen receptor; H&E: hematoxylin and eosin; PR: progesterone receptor

The patient of case 6 showed typical microscopic findings of low-grade adenosarcoma (Fig. 2). Phyllodes-like structures, covered by one layer of benign epithelia with focal mucinous metaplasia, were observed.

### Detailed clinicopathological findings of the recurrent cases

#### Case 1

**Fig. 3:**
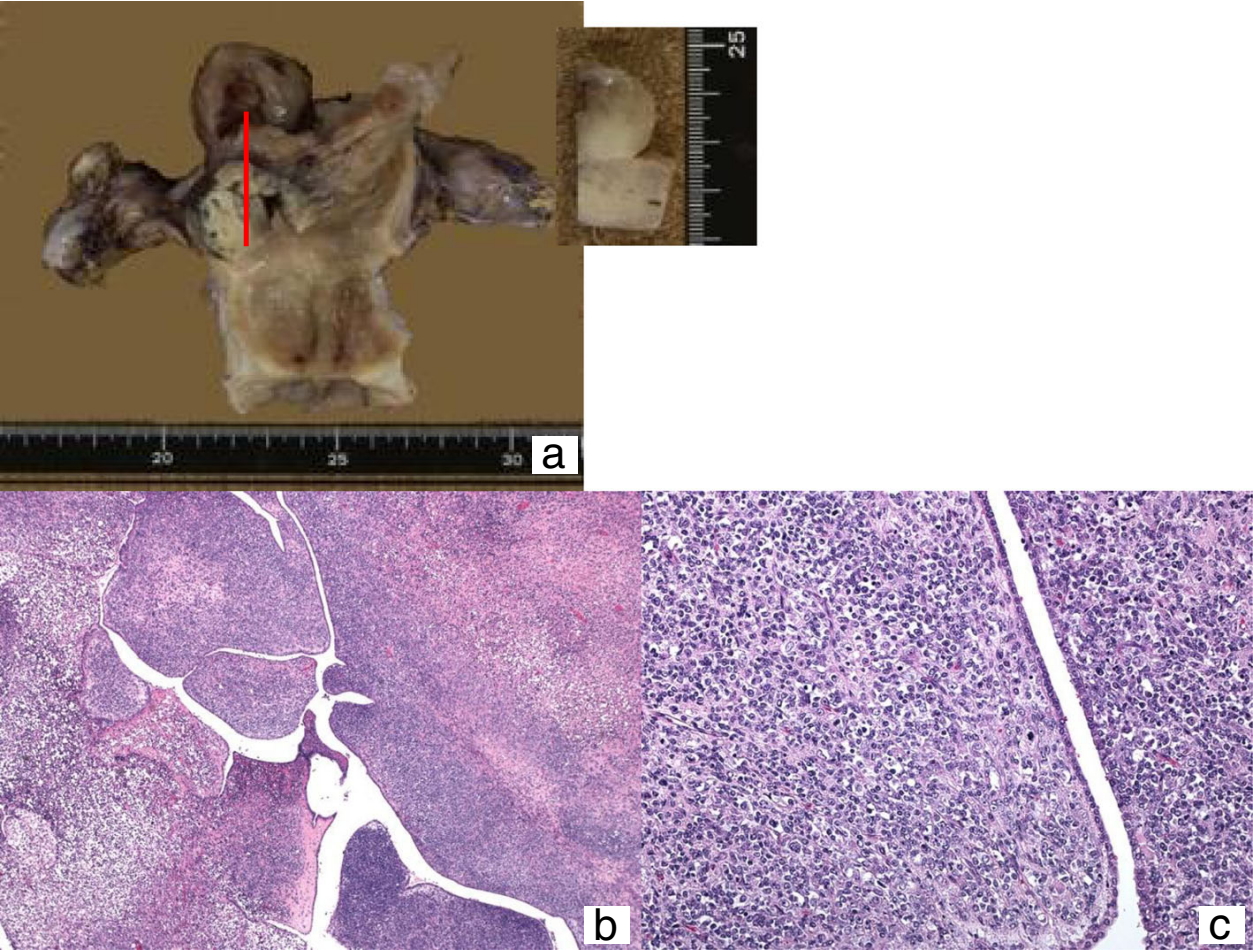
Case 1. (**a**) Macroscopic findings. A polypoid solid tumor arising from the uterine fundus. The base of the tumor is in white color; the surface layer is amber, which suggests degeneration. (**b**) H&E staining (original magnification × 40) (**c**) H&E staining (original magnification × 400). A leaf-like architecture is shown. Atypical short spindle cells show mild polymorphism and comprise the mesenchymal component. It is covered by a benign glandular epithelium, similar to the intimal surface epithelium. H&E: hematoxylin and eosin

The patient was a 51-year-old female who visited our hospital for abnormal bleeding. Magnetic resonance imaging detected a 9-cm tumor filling the uterine cavity and invading through the myometrium. The tumor was diagnosed as sarcoma by biopsy. Hysterectomy and bilateral salpingo-oophorectomy was performed. Macroscopically, a polypoid tumor arising from the uterine fundus extended to the serosa; ascites cytology was positive for malignancy. Microscopically, the highly cellular mesenchymal tumor was covered with benign glandular epithelium similar to the intimal surface epithelium and showed leaf-like architecture. Tumor cells were atypical short spindle cells with small round nuclei with mild polymorphism. Some nuclear mitoses were observed (five mitoses/10 HPF). Tumor necrosis and hemorrhage were present. Lymphovascular space invasions were observed extensively. No heterologous elements were found (Fig. 3).

**Fig. 4:**
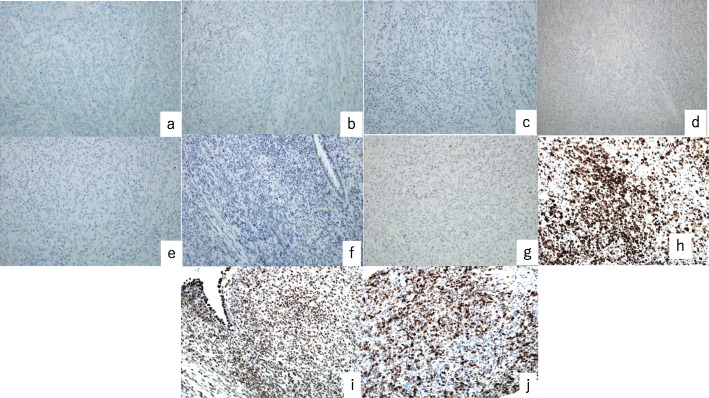
Case 1. (**a**–**j**) Immunohistochemical staining is negative for (**a**) ER, (b) PR, (**c**) CD10, (**d**) cyclin D1, (**e**) cytokeratin, (**f**) BCOR, and (**g**) desmin. (**h**) Overexpressed *p53*. Staining is positive for (**i**) SMARCA4 (original magnification × 400). (**j**) The MIB-1 proliferation index is 70%. ER: estrogen receptor; PR: progesterone receptor

Immunohistochemical results of the mesenchymal area were negative for ER, PR, CD10, cyclin D1, cytokeratin, BCOR, desmin, SMA, CD34, and S100; p53 was overexpressed; SMARCA4 expression was not lost; and the MIB-1 proliferation index was 70% (Fig. 4).

**Fig. 5:**
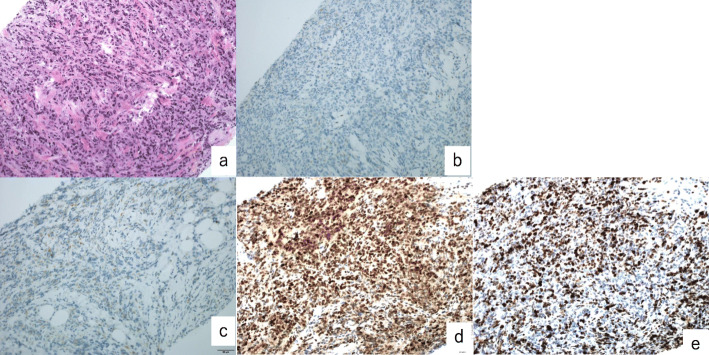
Case 1. (**a**–**d**) Recurrent tissue biopsy of the retroperitoneal mass. (**a**) H&E staining. A mesenchymal component the same as the primary tumor without the epithelial component is observed. (**b**) ER and (**c**) PR are negative, and (**d**) p53 shows overexpression. (**e**) The MIB-1 proliferation index is 80% (original magnification × 400). ER: estrogen receptor; H&E: hematoxylin and eosin; PR: progesterone receptor

The patient underwent six cycles of adjuvant chemotherapy (ifosfamide, epirubicin, and cisplatin) but the tumor recurred in the retroperitoneal cavity 6 months after surgery. Biopsy of the recurrent tumor showed pure sarcoma, the same as the primary tumor without an epithelial component. The immunohistochemical results were negative for ER, PR, CD10, and SMA; p53 was overexpressed; and the MIB-1 proliferation index was 80% (Fig. 5). After one cycle of chemotherapy (docetaxel and gemcitabine) the recurrent mass progressed and infiltrated the ureter. The patient died of disease 1 month after the primary surgery.

#### Case 2

**Fig. 6:**
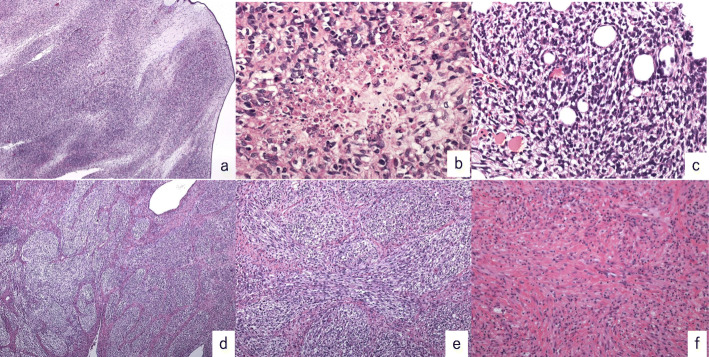
Case 2. Primary site. (**a**) H&E staining (original magnification × 40) (**b**) H&E staining (original magnification × 400). Circular shapes or short spindle cells are multiplied sparsely around the necrosis tissue. Brisk mitotic activity and atypical cells are observed. (**c**) Recurrent site H&E staining in iliac bone biopsy showing the same sarcoma component as the primary site (original magnification × 400). Case 3. Primary site. (**d**) H&E staining (original magnification × 40) (**e**) H&E staining (original magnification × 400). Inside the leaf-like structure covered by a layer of flattened epithelium, clear cytoplasm cells constitute a multi-nodular structure. (**f**) Recurrent site H&E staining shows spindle cells and abundant foam cells (original magnification × 400). ER: estrogen receptor; H&E: hematoxylin and eosin; PR: progesterone receptor

The patient was a 62-year-old female who presented to our hospital with massive genital bleeding. A large mass was protruding through the cervix, and the patient was diagnosed as having sarcoma by biopsy. Hysterectomy, bilateral salpingo-oophorectomy, omentectomy, pelvic, and paraaortic lymphadenectomy were performed. The tumor developed from the posterior wall of the uterine corpus, slightly invading the myometrium. There was no lymphovascular space invasion or lymph node metastasis. Microscopically, mostly circular-shaped cells were observed multiplied sparsely around the necrosis tissue. In some areas, brisk mitotic activity, up to 10–15/10 HPF, were found (Fig. 6a, b). These areas were considered as sarcomatous overgrowth. The sarcoma component showed non-specific mesenchymal neoplastic features without heterologous elements. Immunohistochemical tests were negative for ER, PR, S100, CD34, cytokeratin, HHF35, and SMA; positive for SMARCA4, BCOR, and CD10; p53 expression had a wild-type pattern; and the MIB-1 proliferation index was 50%. The patient had recurrences to multiple bones and pulmonary metastasis at 6 years after surgery. Biopsy of the iliac bone showed pure sarcoma, the same as the primary tumor without an epithelial component (Fig. 6c). Immunohistochemical tests of the recurrence tumor were the same as the primary tumor, except for the p53 overexpression. The patient died of disease 2 months after recurrence.

#### Case 3

The patient was a 66-year-old female who presented to our hospital with abnormal bleeding and a 9-cm mass partially infiltrated into the uterine myometrium and occupying the uterine cavity. Hysterectomy, bilateral salpingo-oophorectomy, omentectomy, and pelvic and paraaortic lymphadenectomies were performed. Inside the leaf-like structure covered by one layer of flattened epithelium, cells with a clear cytoplasm constituted a multi-nodular structure (Fig. 6d, e). The immunohistochemical results were negative for ER, PR, cyclin D1, and BCOR; positive for HHF35, SMA, desmin, and SMARCA4; p53 was overexpressed; and the MIB-1 proliferation index was 50%. The sarcomatous component was considered to show leiomyomatous differentiation. No heterologous elements were observed. A 40-mm tumor appeared in the S1 region of the lung 1 year after surgery. Biopsy of the recurrent tumor showed spindle cells and abundant foam cells comparable with the state after chemotherapy (Fig. 6f). Immunohistochemical tests of the recurrence tumor were the same as the primary tumor. The pulmonary tumor responded to two cycles of chemotherapy (ifosfamide, doxorubicin, and cisplatin) and upper lobe resection was performed; four more cycles of chemotherapy were performed after operation. The patient died of a subarachnoid hemorrhage due to rupture of a cerebral aneurysm 5 months after the last chemotherapy treatment.

## Discussion

All seven patients aged 40 years-old at diagnosis and six of them were menopausal. All patients presented with polypoid tumors and abnormal bleeding, and had FIGO stage I disease with myometrium invasion. Four patients experienced recurrence and had sarcomatous overgrowth, with negative ER and PR; three patients had high MIB-1 proliferation ratio and p53overexpression. Three patients had recurrences in distant sites and biopsied tissues were pure sarcoma with the same immunohistochemical staining patterns as the primary tumor; in one case p53 expression became significantly stronger in the recurrent tumor compared with the primary. Two patients with recurrence were not adapted for surgery, progressed rapidly, and died 1–2 months after recurrence.

Adenosarcoma was initially described by Clement and Scully in 1974 [13], who described the tumor as an “admixture of a sarcomatous stromal element resembling endometrial stromal sarcomas of various types and grades and a benign, but often atypical, epithelial component.” The sarcomatous component is usually a low-grade uterine sarcoma, mostly endometrial stromal sarcoma, and the epithelium is often endometrium-like cells. Mixed tumors of epithelial and mesenchymal components are diagnosed as adenofibroma, adenosarcoma, and carcinosarcoma according to whether each component is benign or malignant. When the sarcomatous component occupies more than 25% of the tumor, it is defined as sarcomatous overgrowth.

Adenosarcomas arise mainly from endometrial lesions (85%), with others arising from the cervix, or being extrauterine (e.g., ovary, peritoneum, vagina) [1]. A mass protruding from the external os and occupying the uterine cavity and abnormal vaginal bleeding are the most common symptoms.

Only 3.1% and 2.5% of patients had nodal and/or distant metastases at diagnosis and stage 1 cancer without sarcomatous overgrowth have a good prognosis, which is defined as 5-year overall survival rate of 80% [1]. However, the following pathological findings are reported to be poor prognostic factors: sarcomatous overgrowth, myometrial invasion, lymphatic and/or vascular invasion, heterologous elements of the sarcomatous component (rhabdomyosarcomatous differentiation), extrauterine spread, and an extrauterine origin [3–6]. Recurrence intervals were 0.5–9.5 years after primary surgery. Approximately half of the recurrent tumors were pure sarcomas while the others were adenosarcomas. Most of the recurrent tumors were typically less well-differentiated than the primary tumors, having non-specific spindle-cell sarcomas, with higher mitotic rates [2].

Our patients’ ages at diagnosis and presentation of polypoid tumor with abnormal bleeding were typical for the clinical course of adenosarcomas. Most recurrences of adenosarcomas occur locally, such as in the vagina and pelvis, and distant metastases are described in approximately 5% of cases [2]. Our patients had hematogenous recurrence in distant sites; nevertheless, they were all primarily in the early stage. Pathologically, all four patients with recurrent tumors showed high-grade sarcoma features, such as marked nuclear atypia, coarse chromatin, prominent nucleoli, pleomorphism, weak leaf-like structures, and sarcomatous overgrowth. Immunohistochemical staining showed negative results for ER and PR, high MIB-1 proliferation, and three patients p53 was overexpressed. Taken together, these findings suggested that the four patients with recurrent adenosarcomas had sarcoma components with high-grade malignant potential at the onset.

Pathological diagnosis of adenosarcomas is based on the histomorphological observation of the tissue specimens with hematoxylin and eosin staining. As the morphological analyses of adenosarcomas do not always meet typical findings, immunohistochemical tests have also been used. The most common immunohistochemical markers for the sarcomatous component of adenosarcomas are CD10 (71–100%), WT1 (79%), and vimentin (86%) [7, 8]. CD10 positivity has been found to be lower in patients with sarcomatous overgrowth [14]. Other markers for adenosarcomas are SMA (50–68%), desmin (32–62.5%), CD34 (35%), and cytokeratin (25–27%). In our study, the primary tumors positive for CD10, SMA, desmin, CD34, and cytokeratin were 60% (3/5), 33% (1/3), 40% (2/5), 50% (1/2), and 0% (0/3), respectively.

Adenosarcomas frequently expresses hormone receptors (ER, PR, and androgen receptors) similar to endometrial stromal cells or tumors. However, adenosarcomas with sarcomatous overgrowth lose expression of the hormone receptors, reflecting the nature of dedifferentiation [7, 8, 15]. Tasaka et al. [16] reported an adenosarcoma case arising from endometriosis. The primary tumors were positive for ER and PR, without sarcomatous overgrowth; however, the tumors’ regrowth revealed sarcomatous overgrowth with reduced expression levels of the hormone receptors. Estrogen stimulation by, for example, tamoxifen [17], pre-existing Stein-Leventhal syndrome [2], or ovarian thecoma [18], may play a role in the development of adenosarcoma. There are few cases reporting on hormone therapy [19, 20]; however, whether hormone receptors are prognostic factors for response to hormone therapy has not been determined yet.

*TP53* is a well-known tumor suppressor gene, and the p53 protein expression measured by immunohistochemistry is highly correlated with the mutation status. In normal cells, p53 protein stains weakly in a heterogeneous fashion (wild-type). In constrast, in p53-mutated cells, p53 stains strongly in a homogenous fashion (overexpression) or is completely lost (null pattern). *TP53* mutations have been reported only in a small fraction of adenosarcomas. Recently, Hodgson et al. [9], performed comprehensive genomic analysis targeting exons of 409 oncogenes and tumor suppressor genes of nine high-grade adenosarcomas. High-grade adenosarcomas are frequently associated with *TP53* pathway alterations, identified in 7/9 (78%) cases. High-grade adenosarcoma with p53 protein overexpression might be aggressive with short-interval recurrences and metastases, regardless of sarcomatous overgrowth.

In our study, three recurrent patients showed p53overexpression in the primary tumors; One patient showed a wild-type expression pattern in the primary tumor, which became overexpressed at recurrence. Patients with recurrence had poor prognoses, consistent with previous reports. Whether expression of p53 changes at recurrence remains unclear.

We also performed SMARCA4 staining, for which none of our case showed a loss of expression. Loss of SMARCA4 by immunohistochemistry indicates biallelic inactivation of the *SMARCA4* genes [21]. The *SMARCA4* gene is one of the tumor suppressor genes that encode the BRG1 protein, a subunit of the SWI/SNF complex. SMARCA4 loss leads to extremely aggressive malignancy in young patients, such as ovarian small cell carcinomas of the hypercalcemic type [22] and thoracic sarcoma [23]. Recent genomic studies have identified loss of SMARCA4 in undifferentiated uterine sarcomas and dedifferentiated carcinomas of the endometrium or ovary [10, 24]. However, there have been no reports showing an association between adenosarcomas and SMARCA4 loss. In this research, the positive immunostaining results for SMARCA4 suggested that its inactivation was not associated with tumorigenesis of adenosarcoma.

*BCOR* is another SWI/SNF component gene that might affect high-grade sarcomas in young patients, such as clear cell sarcomas of kidney. Marino-Enriquez et al. [12] reported cases of patients aged 18–32 years with three high-grade sarcomas with BCOR, regarded as a unique subtype, possibly within the family of endometrial stromal neoplasia. Muthukumarana et al. [11] performed BCOR immunohistochemistry for 13 adenosarcomas and reported that nine cases expressed BCOR regardless of sarcomatous overgrowth. However, only one of these BCOR-positive cases harbored a *BCOR* gene rearrangement as determined by fluorescent in situ hybridization. In our research, one patient was positive for BCOR; however, further analysis of BCOR rearrangements should be performed in adenosarcomas that demonstrate BCOR expression.

The small study sample and the fact that it was obtained from only one institute was a limitation of this study. More multicenter cases and further molecular biology studies are awaited.

## Conclusion

To the best of our knowledge, this is the first adenosarcoma case series report comparing microscopic and immunohistochemical findings of primary and recurrent tumors. Most findings that relate to the high-grade malignant potential of the sarcoma component were essentially the same between the primary and recurrent tumors, suggesting that the initial pathological observation of the primary tumor is important for the assessment of the prognosis. Malignant transformation of adenosarcomas might occur through additional mutation of the *p53* gene. Molecular testing, including for SMARCA4 and BCOR, might not be useful for predicting prognoses of adenosarcomas.

## Data Availability

All data generated or analyzed during this study are included in this published article.

## Abbreviations

AWD: Alive with disease
BSO: Bilateral salpingo-oophrectomy
DOD: Death of disease
ER: Estrogen receptor
HPF: High power field5-FU: 5-fluorouracil
LVSI: Lymphovascular invasion
mRH: Modified radical hysterectomy
NED: No evidence of disease
ND: Not done
PALA: Paraaortic lymphadnectomy
PLA: Pelvic lymphadnectomy
PR: Progesterone receptor
SAH: Subarachnoid hemorrhage
SMA: Smooth muscle antibody
SO: Sarcomatous overgrowth
TAH: Total abdominal hysterectomy
